# Mirabegron for overactive bladder in frail patients 80 years or over (HOKUTO study)

**DOI:** 10.1186/s12894-022-00989-7

**Published:** 2022-03-21

**Authors:** Hiroshi Nakagomi, Takahiko Mitsui, Hiroshi Shimura, Tatsuya Ihara, Satoru Kira, Norifumi Sawada, Masayuki Takeda

**Affiliations:** Department of Urology, University of Yamanashi Graduate School of Medical Sciences, 1110 Shimokato, Chuo City, Yamanashi 409-3898 Japan

**Keywords:** OAB, β3-adrenoceptor agonist, Older, Frail, Older society

## Abstract

**Background:**

We assessed the efficacy and safety of mirabegron, a β_3_-adrenoceptor agonist, in older adults (≥ 80 years old) with overactive bladder (OAB).

**Methods:**

OAB patients aged ≥ 80 years were enrolled in this prospective, single-arm observational study. OAB was diagnosed based on the OAB symptom score (OABSS); i.e., a total score of ≥ 3 points and an urgency score of ≥ 2 points. Patients who received 50 mg mirabegron once daily were evaluated at the baseline and at 4, 8, and 12 weeks. The changes from the baseline in the OABSS, International Prostate Symptom Score (IPSS), OAB questionnaire (OAB-q) score, and Vulnerable Elders Survey (VES-13) score were determined. Adverse events, laboratory tests, 12-lead electrocardiography, the QT interval according to Fridericia’s formula (QTcF), uroflowmetry, the post-void residual urine volume (PVR), and the Mini-Mental State Examination (MMSE) score were used to assess safety.

**Results:**

Forty-three patients (median age: 84 years, range: 80–96 years) were examined. They had high rates of comorbidities and polypharmacy. Mirabegron significantly improved in total score of the OABSS, including urgency and urge incontinence. The total IPSS, IPSS quality-of-life (QOL) index, and OAB-q scores also significantly improved. Mirabegron improved in the VES-13 score. There were no significant changes in laboratory test values, uroflowmetry findings, PVR, the QTcF, or MMSE score. Two patients (4.7%) withdrew from the study after experiencing adverse events.

**Conclusions:**

Mirabegron was well tolerated and significantly improved in OAB symptoms, and QOL in older patients.

*Trial registration* The present clinical study was approved by University of Yamanashi Institutional Review Board prior to study initiation (ID1447) and was retrospectively registered with the UMIN Clinical Trials Registry (UMIN-CTR), Japan (UMIN000045996) on Nov 6, 2021.

**Supplementary Information:**

The online version contains supplementary material available at 10.1186/s12894-022-00989-7.

## Background

The number and proportion of older people is increasing all over the world. In Japan, the proportion of the population aged > 65 will reach almost 30% in the near future. Since overactive bladder (OAB) is common in aged people and has a major influence on quality of life (QOL), it is important to diagnose and treat OAB in older societies. Currently, anticholinergics and β_3_-adrenoceptor agonists are the main treatments for OAB. Focusing on the age of clinically treated OAB patients, a study of the treatment of OAB based on Japanese prescription databases revealed that the mean age of the patients was 74.0 years old (y/o), and their median age was around 80 y/o. Furthermore, 81.3% of OAB patients that received prescriptions for treatment were ≥ 65 y/o, and 59.4% were ≥ 75 y/o [1].

In older patients, frailty is currently a major global health burden in older societies because it results in negative health outcomes [2]. Frailty is also known to be associated with OAB [3, 4]. Thus, it is an important factor in the treatment of older patients with OAB. Furthermore, anticholinergics are known to cause some adverse events (AEs), particularly cognitive function impairment, in older patients. On the other hand, it is well known that β3-adrenoceptor agonists have different mechanisms of action to anticholinergics and do not cause the typical AEs associated with anticholinergics. However, in older patients there is little evidence about whether β3-adrenoceptor agonists induce AEs when used to treat OAB.

Mirabegron was developed as the first β3-adrenoceptor agonist for treating OAB in Japan, and it has been widely used to treat OAB, including in older patients. In the present study, the efficacy and safety of mirabegron were assessed in older patients (≥ 80 y/o) with OAB.

## Methods

### Study design

This 12-week, prospective, single-arm observational study (University Hospital Medical Information Network Clinical Trials Registry, ID1447) (UMIN000045996, Nov 6, 2021, retrospectively registered) was conducted between March 2016 and July 2017 at two community-based hospitals (Koyo Hospital and Shiokawa Hospital) in Yamanashi, Japan. The study protocol was approved by the institutional review boards of each participating hospital. All patients gave their written informed consent after receiving a full explanation about the study.

**Fig. 1:**
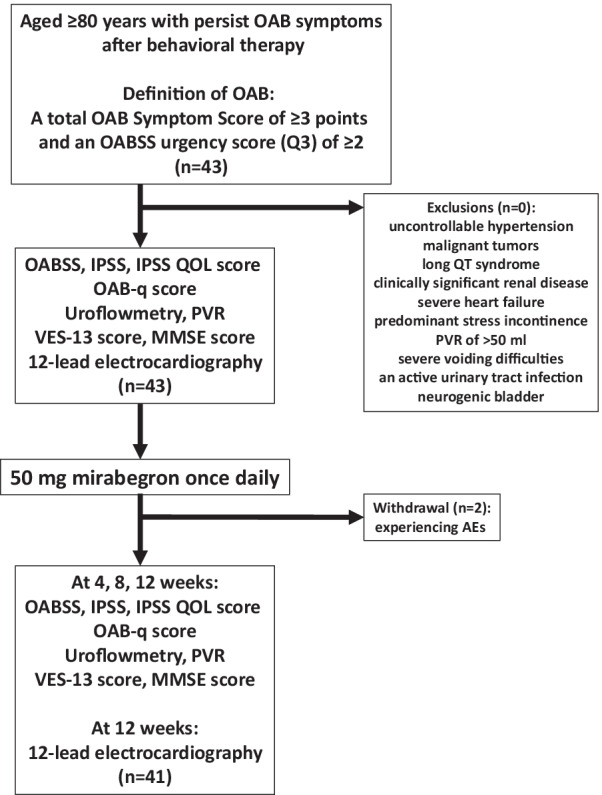
Study flow-chart

Figure 1 showed study-flow chart. Patients aged ≥ 80 years with persist OAB symptoms after behavioral therapy were consecutively enrolled in this study. OAB was defined as a total OAB Symptom Score (OABSS) of ≥ 3 points and an OABSS urgency score (Q3) of ≥ 2 [5]. Patients received 50 mg mirabegron once daily. The examined efficacy endpoints were the changes from the baseline to 4, 8, 12 weeks after treatment in the following items: the OABSS, International Prostate Symptom Score (IPSS), the IPSS QOL score, the OAB questionnaire (OAB-q) score, uroflowmetry (UFM) findings, the post-void residual urine volume (PVR), the Vulnerable Elders Survey-13 (VES-13) score, and the mini-mental state examination (MMSE) score. The VES-13 was used to evaluate frailty in older people. In Japanese, VES-13 scores of 3–6 and ≥ 7 correspond to vulnerable individuals and frail individuals, respectively [6]. The Japanese version of the MMSE was used to measure global cognitive function in the present study [7]. At the baseline and 12 weeks, laboratory tests and 12-lead electrocardiography (ECG) were performed, and the QT interval corrected for heart rate using Fridericia’s formula (QTcF) was also assessed.

Patients were excluded if they had uncontrollable hypertension, malignant tumors, long QT syndrome, clinically significant renal disease, severe heart failure, predominant stress incontinence as determined by the investigator, significant bladder outlet obstruction as indicated by a PVR of > 50 ml, severe voiding difficulties, an active urinary tract infection, neurogenic bladder such as multiple sclerosis or a spinal cord injury who had undergone treatment with other antimuscarinics within the 2 weeks before the baseline, or patients who were judged to be inappropriate subjects by the attending physicians. The continued use of all other medications, including diuretics, alpha-blockers, and 5-alpha-reductase inhibitors, was permitted.

### Efficacy assessments

The changes from the baseline in the OABSS, OAB-q score, IPSS, IPSS QOL score, and VES-13 score seen after treatment were evaluated as efficacy endpoints. The patients were instructed to complete sheets for calculating their OABSS, OAB-q, IPSS, IPSS QOL, and VES-13 scores at their 0-, 4-, 8-, and 12-week visits. The data obtained at the week-0 visit were used as the baseline.

The primary outcome was the changes of total OABSS from the baseline after treatment.

### Safety assessments

Safety was assessed based on AEs, laboratory tests, 12-lead ECG, the QTcF, UFM, the PVR, and the MMSE score. AEs observed after the administration of the first dose of mirabegron were defined as treatment-emergent AEs (TEAEs). UFM findings, the PVR, and the MMSE score were assessed at 0, 4, 8, and 12 weeks. Urinalysis was also performed at these visits. Routine laboratory tests and ECG were conducted at 0 and 12 weeks.

### Statistical analysis

Data are presented as the median and interquartile range (IQR). Changes in the OABSS, IPSS, OAB-q score, VES-13 score, MMSE score, heart rate (HR) and the QTcF were analyzed using analysis of the Wilcoxon signed-rank test. *P*-values of < 0.05 were considered significant. All statistical analyses were performed using JMP pro 16 (SAS institute Inc., NC, USA).

## Results

### Patient characteristics at the baseline

A total of 43 patients were enrolled in this study. Of the enrolled patients, 2 patients (4.7%) withdrew from the study after experiencing AEs.

The patients’ characteristics at the baseline are summarized in Table 1. The median age of the patients was 84 y/o (range: 80–96 y/o). The patients consisted of 15 females (35%) and 28 males (65%). All of the males had benign prostatic hyperplasia (BPH) at baseline and had previously received treatment for BPH with α1-adrenoreceptor antagonists. The patients in this study had a median of 3 (IQR: 2–4) comorbid conditions. The median number of concomitant medications being taken was 6 (IQR: 3-9). The median total OABSS, IPSS, and IPSS QOL scores were 9 (IQR: 8–11), 15 (IQR: 9.5–21), and 5 (IQR: 4–6), respectively. These results indicated that the patients in this study had moderate lower urinary tract symptoms and OAB symptoms. UFM showed a relatively low flow rate without a significant PVR (median 15 ml). The median VES-13 score was 6 (IQR: 3–8), and there were 12 frail patients (28%). The median MMSE score was 27 (IQR: 25–28), and it was ≥ 23 in all but 2 patients (95%) (Table 1).

**Table 1:** Patient characteristics at baseline (all patients (n = 43))

Median age (range)	85 (80–96)
No. of females (%)	15 (35)
No. of males (%)	28 (65)
Median no. of comorbidities (IQR)	3 (2–4)
Median no. of concomitant medications (IQR)	6 (3–9)
Median total OABSS (IQR)	9 (8–11)
Median total IPSS (IQR)	15 (9.5–21)
Median IPSS QOL score (IQR)	5 (4–6)
Median voiding volume (ml) according to UFM (IQR)	106.35 (56.8–132.8)
Median maximum flow rate (ml/s) (IQR)	9.2 (5.4–12.7)
Median PVR (ml) (IQR)	15 (6.5–26.8)
Median VES-13 score (IQR)	6 (3–8)
Median MMSE score (IQR)	27 (25–28)
Median heart rate (beats/min) (IQR)	64 (57–73)
Median QT interval corrected using Fridericia’s formula (ms) (IQR)	419 (397.25–438)

### Efficacy assessments

**Fig. 2:**
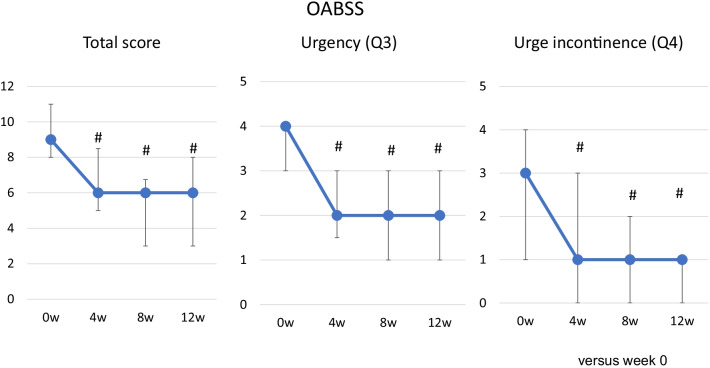
OABSS. Median ± IQR; #*P* < 0.01 (vs. week 0)

**Fig. 3:**
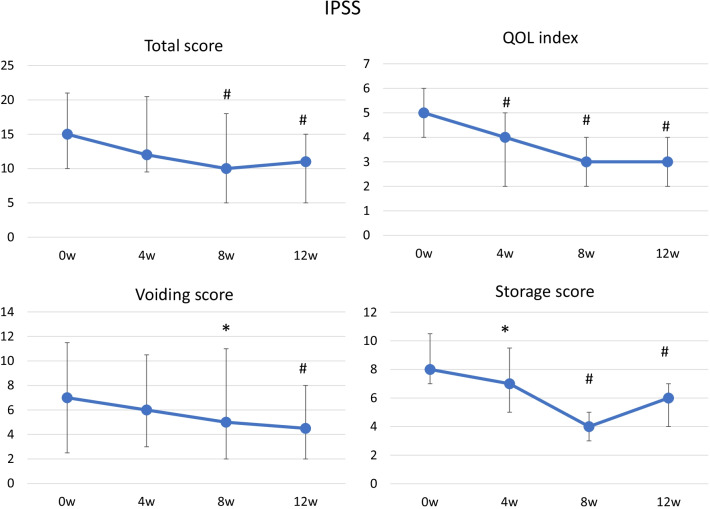
IPSS and IPSS QOL score. Median ± IQR; *P < 0.05; #*P* < 0.01 (vs. week 0)

**Fig. 4:**
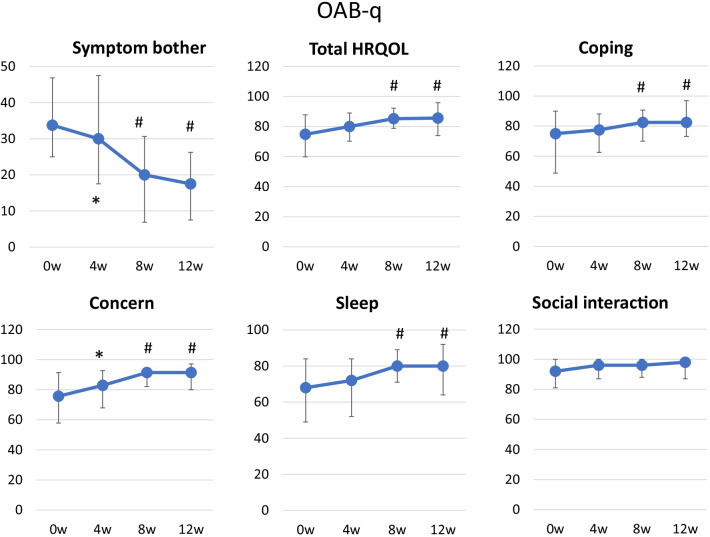
OAB-q score. Median ± IQR; *P < 0.05, #*P* < 0.01 (vs. week 0)

Significant improvements from the baseline were seen in the total OABSS score at 4, 8, and 12 weeks (Fig. 2). The mean change in the total OABSS score between the baseline and week 12 was −3.8 points, and the total OABSS score had normalized by week 12 in 36% (15/41) of the patients. The OABSS scores for urgency and urge incontinence were also significantly decreased after treatment (Fig. 2). Significant improvements in the total IPSS score from the baseline were seen at 8 and 12 weeks. The IPSS storage symptom score and IPSS QOL score were also significantly improved at 4, 8 and 12 weeks. Significant improvement was observed in the IPSS voiding score at 8 and 12 weeks (Fig. 3). Regarding QOL, OAB-q symptom bother and OAB-q total health-related QOL (HRQL) scores were significantly improved at 8 and 12 weeks. In the HRQL subscales, significant improvements in the concern subscale were seen at 4, 8 and 12 weeks and in the coping and sleep subscale at 8 and12 weeks (Fig. 4).

Focused on gender difference, the same trend of improvement was observed after mirabegron treatment (Additional file 1).

### Safety

For all patients, the overall incidence of TEAEs was 4 cases (9.6%), and that of the drug-related TEAEs was 3 cases (7.3%). The 1 patient (2.3%) who died of a ruptured abdominal aortic aneurysm after 12 weeks did not visit the hospital and received a prescription for mirabegron at 12 weeks. The ER doctors judged that there was no association between the ruptured abdominal aortic aneurysm and the mirabegron treatment. The drug-related TEAEs included lower-extremity edema with redness at week 8, pollakiuria at week 8, and a urinary tract infection at week 4. No cases of urinary retention were noted in this study, and no significant changes from the baseline were seen in the mean PVR or flow rate on UFM.

The median change in HR between the baseline and week 12 was 0.5 beats/min, which was not significant. The median change in the QTcF from the baseline to week 12 was 4 ms (Table 2). However, there were no significant change in the QTcF from the baseline. At week 12, absolute QTcF of > 450 ms were noted in 4 patients (9.7%), but there were no patients with absolute QTcF of > 480 ms. Although increases in the QTcF of 30–60 ms from the baseline to week 12 were seen in 2 patients (4.9%), increases of > 60 ms were not seen in any patients. There were no notable changes from the baseline in any laboratory parameter (Table 2).

**Table 2:** Safety after mirabegron treatment (n = 41)

	Baseline	Week 4	Week 8	Week 12
Heart rate (beats/min)	64 (57–73)			63.5 (58–77)
QTcF (ms)	419 (397.25–438)			415 (401.25–433.25)
MMSE	27 (25–28)	26.5 (24.25–29)	28 (26–29)	27 (26–29.5)
VES-13	6 (3–8)	5 (4–7)	4^#^ (2–5.25)	3* (2.25–6)
VES-13 excluding age	3.5 (1–6)	3 (1–6)	1^#^ (1–3)	1^#^ (1–4)

Median (IQR)

^*^P < 0.05; #*P* < 0.01 (vs. baseline)

There was no deterioration in the mean MMSE score at 4, 8, or 12 weeks compared with the baseline (Table 2).

Regarding frailty, there was significant improvement in the VES-13 score between the baseline and 8 or 12 weeks, though no change between the baseline and 4 weeks. When age was excluded as an item, a significant decrease in the VES-13 score from the baseline were also seen at 8 and 12 weeks (Table 2).

## Discussion

The prevalence of OAB is around 10–20% and increases with age [8, 9], and the treatment of OAB in aged patients, particularly frail patients, is an extremely important issue in older societies. In the present study, we demonstrated that a β3-adrenoceptors agonist, mirabegron, is an effective and generally well tolerated treatment for OAB in very old patients. These results provide important evidence that medically complex older individuals with OAB could benefit from treatment with mirabegron. Hence, mirabegron may be a suitable first-line treatment option for OAB in very old patients.

Healthcare systems currently face various problems associated with aged populations. For example, older individuals may be frail; i.e., exhibit age-associated functional decline, which is one of the most challenging issues in older societies. Frailty is characterized by increased risks of mortality, longer hospitalization, falls, impaired cognitive function, and polypharmacy [2]. It is also known that frailty is associated with OAB [3, 4]. Older patients with OAB often have to take many concomitant medications and may have multiple comorbidities. Such comorbidities, which may be caused by falls or fractures, can impair their activity, [10–12]. Indeed, on average the OAB patients in the present study had more than 3 comorbidities and were taking around 6 concomitant medications. The VES-13 scoring system suggested that 12 patients (28%) were frail. In older societies, it is important to treat older patients with OAB efficiently and safely.

Anticholinergics have been used as the first-line treatment for OAB. In a study regarding the anticholinergic burden in the Japanese elderly population, it was demonstrated that anticholinergics were used more often in patients with OAB than in those without OAB, which was largely attributable to the use of antimuscarinics for OAB [13]. However, anticholinergics can cause various AEs, such as a dry mouth, constipation, and impaired cognitive function. These AEs increase in frequency with age [14]. Furthermore, anticholinergics are also prescribed for various diseases in older individuals, which indicates that older patients may be at high risk of AEs, particularly dementia [15]. Indeed, a systematic literature review revealed that long-term (≥ 3 months) treatment with anticholinergics increased the risk of cognitive impairment or dementia compared with the non-use of such drugs [16]. Another study also reported that higher anticholinergic burden levels are associated with increased risks of falls and fractures [17]. Therefore, particularly in older patients, the prescription of anticholinergics should be avoided, and alternative medications, such as β3-adrenoceptor agonists, should be considered for OAB to reduce the anticholinergic burden.

Under these circumstances, pharmacotherapy for OAB should be selected carefully in older patients. Several studies have examined the efficacy and safety of mirabegron in older patients with OAB. In the PILLAR study, which was a phase IV randomized placebo-controlled study investigating the efficacy, safety, and tolerability of mirabegron in OAB patients with incontinence aged ≥ 65 years, mirabegron demonstrated good efficacy, was well tolerated, and had a known safety profile, even in patients aged ≥ 75 years [18–20]. In Japan, Yoshida et al. examined the safety and effectiveness of mirabegron in patients aged ≥ 75 versus those aged < 75 years [20]. Their study revealed that, although compared with the patients aged < 75 years, those aged ≥ 75 years seemed to have more severe OAB, involving a longer disease duration, were frailer (characterized by a lower body mass index), had more comorbidities, and exhibited greater concomitant drug use Thus, mirabegron exhibited good efficacy and tolerability, even in the older patients in a real-world clinical setting.

Griebling et al. revealed that treatment with mirabegron for 12 weeks did not affect cognitive function in patients aged ≥ 65 years, as measured by the Montreal Cognitive Assessment [20]. Welk et al. also reported that the use of mirabegron in patients with OAB was associated with a lower risk of new-onset dementia compared with the use of anticholinergics [21]. Thus, mirabegron is a more suitable treatment for older patients with OAB than anticholinergics in terms of its efficacy, safety, and tolerability.

However, there are still questions about whether mirabegron is effective and safe in older OAB patients because according to Japanese databases the median age of the patients prescribed OAB medications is around 80 y/o [1], which means that there many OAB patients aged ≥ 80 y/o that are prescribed such drugs. Therefore, the present study focused on the efficacy and safety of mirabegron in OAB patients aged ≥ 80 y/o. It revealed that mirabegron is an effective and generally well tolerated treatment for ≥ 80 y/o OAB patients. Furthermore, while mirabegron did not affect the cognitive function of these older OAB patients, it may have reduced their frailty. This may have been due to the OAB patients being able to engage in more social activity. This is the first study to provide important evidence about medical treatment for OAB in older patients.

Our study has several limitations. First, the results are preliminary because this was a small and single-arm open-label trial conducted in Japan. An additional placebo-controlled study would be necessary to elucidate the true efficacy profile of mirabegron in older OAB patients. Second, to evaluate frailty, we used the VES-13, which may not accurately assess frailty. Third, the MMSE may not be sufficiently sensitive for detecting small short-term changes in cognitive function. Fourth, any further data on safety and efficacy at longer follow-up of more than 12 weeks are lacking. Further studies are necessary to examine these points.

## Conclusions

Mirabegron significantly ameliorated OAB symptoms and was generally well tolerated in older patients. It also significantly improved QOL in these patients. This study provided very important evidence regarding the treatment of OAB in older patients.

## Supplementary Information

**Additional file 1:** Gender difference in IPSS, OABSS and OAB-q.

## Data Availability

We cannot provide and share our datasets in publicly available repositories because of informed consent for participants as confidential patient data.

## Abbreviations

AEs: Adverse events
ECG: Electrocardiography
HR: Heart rate
HRQL: Health-related QOL
IPSS: International Prostate Symptom Score
IQR: Interquartile range
MMSE: Mini-Mental State Examination
OAB: Overactive bladder
OABSS: Overactive Bladder Symptom Score
OAB-q: OAB questionnaire
PVR: Post-void residual urine volume
QOL: Quality of life
QTcF: QT corrected for heart rate using Fridericia’s formula
TEAEs: Treatment-emergent adverse events
UFM: Uroflowmetry
VES-13: Vulnerable Elders Survey
y/o: Years old
